# The brassinosteroid receptor gene *BRI1* safeguards cell-autonomous brassinosteroid signaling across tissues

**DOI:** 10.1126/sciadv.adq3352

**Published:** 2024-09-25

**Authors:** Noel Blanco-Touriñán, Surbhi Rana, Trevor M. Nolan, Kunkun Li, Nemanja Vukašinović, Che-Wei Hsu, Eugenia Russinova, Christian S. Hardtke

**Affiliations:** ^1^ Department of Plant Molecular Biology, University of Lausanne, Lausanne, Switzerland.; ^2^ Department of Biology, Duke University, Durham, NC, USA.; ^3^ Howard Hughes Medical Institute, Duke University, Durham, NC, USA.; ^4^ Department of Plant Biotechnology and Bioinformatics, Ghent University, 9052 Ghent, Belgium.; ^5^ Center for Plant Systems Biology, VIB, Ghent, Belgium.

## Abstract

Brassinosteroid signaling is essential for plant growth as exemplified by the dwarf phenotype of loss-of-function mutants in *BRASSINOSTEROID INSENSITIVE 1* (*BRI1*), a ubiquitously expressed Arabidopsis brassinosteroid receptor gene. Complementation of brassinosteroid-blind receptor mutants by *BRI1* expression with various tissue-specific promoters implied that local brassinosteroid signaling may instruct growth non–cell autonomously. Here, we performed such rescues with a panel of receptor variants and promoters, in combination with tissue-specific transgene knockouts. Our experiments demonstrate that brassinosteroid receptor expression in several tissues is necessary but not sufficient for rescue. Moreover, complementation with tissue-specific promoters requires the genuine *BRI1* gene body sequence, which confers ubiquitous expression of trace receptor amounts that are sufficient to promote brassinosteroid-dependent root growth. Our data, therefore, argue for a largely cell-autonomous action of brassinosteroid receptors.

## Teaser

Ubiquitously expressed trace amounts of BRI1 are necessary and sufficient for brassinosteroid-dependent growth regulation.

## INTRODUCTION

Brassinosteroids, such as the prototypical brassinolide, are endogenous key regulators of plant growth (*1*, *2*). In Arabidopsis (*Arabidopsis thaliana*), brassinolide is sensed by the extracellular domains of the receptor kinases BRASSINOSTEROID-INSENSITIVE 1 (BRI1) and its homologs BRI1-LIKE 1 (BRL1) and BRI1-LIKE 3 (BRL3) (*3*–*5*). Brassinolide binding promotes their interaction with co-receptors, triggering a phospho-transfer cascade that permits nuclear accumulation of downstream transcription factors to regulate target genes (*6*–*9*). The numerous genes controlled by brassinosteroid signaling include brassinosteroid biosynthesis pathway genes, establishing a feedback loop that maintains brassinosteroid signaling homeostasis. Loss-of-function mutations in *BRI1* or genes encoding key brassinosteroid biosynthetic enzymes lead to severe growth retardation, including strongly impaired root growth (*1*, *10*–*15*). The root phenotype of brassinosteroid-related mutants presents various aspects (*14*). Although cells are generally shorter, this does not scale with the overall reduction in root growth because of the additional impact of brassinosteroid signaling on cell proliferation (*11*, *12*, *16*). Furthermore, brassinosteroid signaling restricts formative divisions, mostly in the stele (*13*, *17*–*19*), and is also required for the proper formation of vascular tissues (*3*, *13*, *20*–*22*), which are, in turn, important for root growth maintenance (*23*, *24*). Recent morphometric three-dimensional (3D) single-cell analyses found that brassinosteroid signaling promotes cellular anisotropy but is not required for volumetric cell growth (*17*, *18*) and also enforces accurate cell division plane orientation (*18*). Moreover, single-cell RNA sequencing (scRNA-seq) suggests that overall specification and development of the different root tissue layers progresses correctly in brassinosteroid receptor mutants (*18*, *25*). Thus, their reduced root growth can be parsimoniously explained by the combined effects of reduced cellular anisotropy and aberrant cell divisions (*13*, *17*, *26*). These effects may be aggravated by brassinosteroid-dependent inter–cell layer communication (*17*, *27*), which is also a salient feature of wild-type root development because rate-limiting brassinosteroid biosynthesis genes are expressed in specific cell layers along a spatiotemporal gradient (*28*). Differential biosynthesis may thus be responsible for differential brassinosteroid effects along the root, with lower levels favoring meristematic activity and higher levels favoring cellular anisotropy (*28*). Moreover, brassinosteroid may thereby also coordinate the differential growth dynamics and cell geometry of the vascular cylinder tissues as compared to surrounding tissues (*13*, *17*, *18*, *27*).

**Fig. 1.**
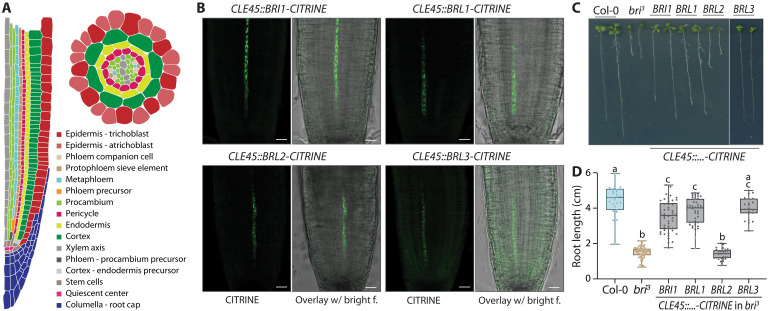
Arabidopsis brassinosteroid receptors are interchangeable in complementation of brassinosteroid-blind mutants with tissue-specific promoters. (**A**) Schematic presentation of an Arabidopsis root tip with a (half) longitudinal and a cross section. (**B**) Confocal microscopy images of root meristems from indicated genotypes (without counterstaining), showing CITRINE signal of indicated fusion proteins (green fluorescence, left) and their overlay with bright-field images (right). (**C**) Representative 9-day-old Col-0 wild-type, *bri^3^* triple-mutant, and transgenic *bri^3^* seedlings expressing indicated brassinosteroid receptor CITRINE fusion proteins under control of the *CLE45* promoter. (**D**) Root growth quantification for 8-day-old seedlings of the indicated genotypes. Box plots display second and third quartiles and the median; bars indicate maximum and minimum. Statistically significant differences (lowercase letters) were determined by ordinary one-way analysis of variance (ANOVA), *P* < 0.004. Scale bars, 20 μm (B) and 1 cm (C).

The precisely aligned cell files that differentiate into the distinct root tissues are produced by apical stem cells at the root meristem tip and give rise to a stereotypic pattern of radial symmetry in the outer tissue layers and bilateral symmetry in the vascular cylinder (Fig. 1A). Intriguingly, although *BRI1* is expressed throughout the root (fig. S1), the phenotype of *bri1* mutants can be complemented by transgenic expression of BRI1 under control of tissue-specific promoters that confer expression in epidermal or vascular tissues (*12*, *13*, *27*, *29*). This could also be observed in *bri1 brl1 brl3* (*bri^3^*) triple receptor null mutants (*13*, *27*) in which aspects of the *bri1* phenotype are aggravated (*3*, *13*, *30*), ruling out compensatory effects by *BRL1* or *BRL3* that are expressed at much lower levels than *BRI1* (*3*, *25*) (fig. S1). To some degree, the extent of complementation depends on the tissue and the expression level. For example, excess epidermal BRI1 mimics brassinosteroid signaling gain-of-function effects (*12*, *27*, *31*), whereas high BRI1 levels in the vascular cylinder can trigger supernumerary formative divisions (*13*, *17*, *18*, *29*). Also, more restricted *BRI1* expression in the two developing protophloem sieve element cell files can largely restore *bri^3^* root growth but confers an intermediate rescue of cellular features (*13*, *18*, *26*). One key conclusion from these experiments is that brassinosteroid receptors may instruct growth non–cell autonomously (*12*, *13*, *26*, *27*, *29*, *31*, *32*).

Here, we sought to determine the nature of the proposed non–cell-autonomous *BRI1* effects. We found that the *BRI1* gene promotes its own ubiquitous expression through gene body–intrinsic sequences and that trace amounts of BRI1 across multiple tissues are necessary to complement *bri^3^* mutants. Thus, our data suggest that the brassinosteroid receptor acts largely cell autonomously.

## RESULTS

### Brassinosteroid receptor genes are interchangeable in *bri*^*3*^ rescue by phloem-specific expression

We first evaluated whether *BRI1* homologs can reproduce the reported *bri^3^* rescue observed upon expression of BRI1-CITRINE fusion protein with the phloem-specific *COTYLEDON VASCULAR PATTERN 2* (*CVP2*), *BARELY ANY MERISTEM 3* (*BAM3*), and *MEMBRANE-ASSOCIATED KINASE REGULATOR 5* (*MAKR5*) promoters (*13*, *26*). Because we previously found that the extent of rescue by *CVP2::BRI1-CITRINE* depends on transgene expression level and dosage (*26*), we expressed the receptor fusion proteins using the promoter of the protophloem sieve element–specific gene, *CLAVATA3/EMBRYO SURROUNDING REGION 45* (*CLE45*) (*33*, *34*), which is expressed at higher levels than *CVP2* (fig. S1). Substantial *bri^3^* rescue was observed with BRI1, BRL1, and BRL3 but not with the related (nonbrassinosteroid) receptor BRL2 (Fig. 1, B to D). Consistent with previous results (*3*, *30*), our findings reiterate that BRI1, BRL1, and BRL3 are functionally equivalent in their ability to rescue the root growth of *bri^3^* when expressed at similar levels.

### Brassinosteroid signaling in multiple tissues is necessary for comprehensive *bri*^*3*^ rescue

**Fig. 2.**
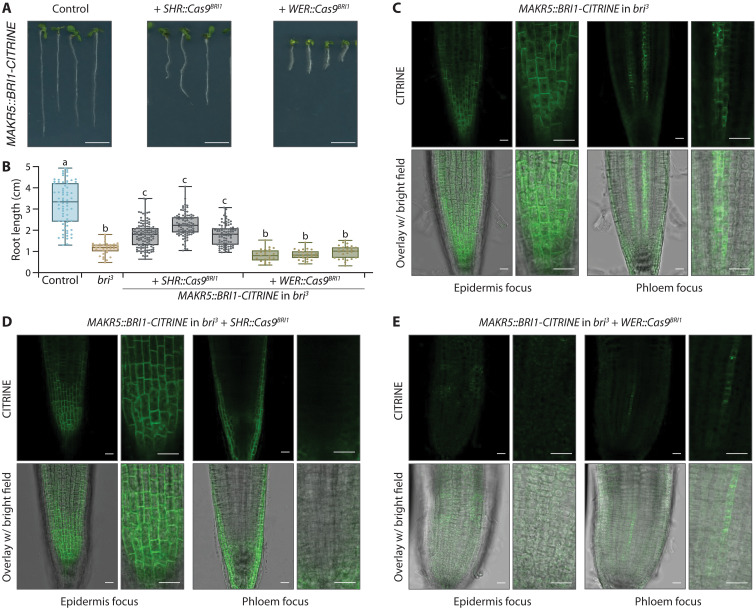
Brassinosteroid perception in both phloem and epidermis is necessary for comprehensive rescue of ***bri^3^*** mutants. (**A**) Representative 8-day-old *bri^3^* seedlings complemented with a *MAKR5::BRI1-CITRINE* transgene (left) and the same line combined with tissue-specific CRISPR-Cas9 *BRI1* knockout using the *SHR* (middle) or *WER* (right) promoters. (**B**) Root growth quantification for 8-day-old seedlings of the indicated genotypes; three independent CRISPR-Cas9 knockout lines are shown. Box plots display second and third quartiles and the median; bars indicate maximum and minimum. Statistically significant differences (lowercase letters) were determined by ordinary one-way ANOVA, *P* < 0.001. (**C** to **E**) Confocal microscopy images of root meristems from indicated genotypes imaged without any counterstaining. BRI1-CITRINE signal (green fluorescence) is observed in the epidermis although the *MAKR5* promoter is phloem pole specific (C). CRISPR-Cas9 knockout using either the stele-specific *SHR* or the epidermis-specific *WER* promoter leads to disappearance of phloem pole (D) or epidermal signal (E), respectively. Scale bars, 20 μm (microscopy images) and 1 cm (seedling images).

The epidermis is a key tissue for brassinosteroid perception (*12*, *32*), and consistently tissue-specific CRISPR-Cas9–mediated *BRI1* transgene knockout in the epidermis using the *WEREWOLF* (*WER*) promoter reverts the phenotype of complemented *bri1* single mutants (*25*). This contrasts with *bri^3^* rescue by expression of brassinosteroid receptors with phloem-specific promoters. To directly test the impact of phloem-specific BRI1 dosage on *bri^3^* rescue, we expressed Cas9 under the control of the *SHORT ROOT* (*SHR*) promoter together with *BRI1*-specific single guide RNAs (gRNAs) (*25*) (*SHR::Cas9^BRI1^*), in a *CVP2::BRI1-CITRINE* reference line that contained three concatenated transgenes in a single locus (*26*). *SHR* is expressed in the vascular cylinder except in the developing protophloem (fig. S1) (*13*, *35*). However, because the *SHR* promoter is active in all vascular stem cells including phloem precursors (*13*, *35*), we could recover transformants in which BRI1-CITRINE signal was no longer detectable in the phloem (fig. S2A). These plants did not display growth defects to the same extent as *bri^3^* (fig. S2B). To corroborate this finding, we transformed the *SHR::Cas9^BRI1^* construct into another homozygous *bri^3^* rescue line that expressed BRI1-CITRINE under the control of the phloem pole–specific *MAKR5* promoter (fig. S1) (*33*). In the progeny of several independent lines that segregated the *SHR::Cas9^BRI1^* transgene, we could compare siblings in which the phloem pole BRI1-CITRINE signal was present with those in which it was absent. Again, the latter did not display a bona fide *bri^3^* phenotype (Fig. 2A); however, compared to their siblings, root growth was reduced to an intermediate length by on average ~31% (Fig. 2B and fig. S2C). The *MAKR5::BRI1-CITRINE* rescue lines also displayed faint yet readily detectable plasma membrane–localized fluorescent signal in the epidermal tissues that was distinct from background fluorescence (Fig. 2C) and persisted in the presence of the *SHR::Cas9^BRI1^* transgene (Fig. 2D and fig. S2, D and E). To determine whether this signal originated from the *MAKR5::BRI1-CITRINE* transgene and whether it had an impact on root growth, we also transformed a *WER::Cas9^BRI1^* construct. *WER* is expressed in epidermal tissues (fig. S1) (*25*, *36*). In the progeny of the *WER::Cas9^BRI1^* plants, we frequently observed loss of the epidermal signal, indicating that it indeed originated from the *MAKR5::BRI1-CITRINE* transgene (Fig. 2E and fig. S2F). Moreover, in such seedlings, root growth complementation was lost, and the plants resembled *bri^3^* mutants (Fig. 2, A and B, and fig. S2C) despite the continued presence of BRI1-CITRINE signal in the phloem poles (Fig. 2E). Collectively, these results indicate that although the phloem contributes to brassinosteroid-mediated root growth, additional low levels of BRI1-CITRINE expression in the epidermis are accountable for the comprehensive rescue of *bri^3^* mutants.

### *BRI1* transgenes display weak epidermal signal independent of tissue-specific promoters

**Fig. 3.**
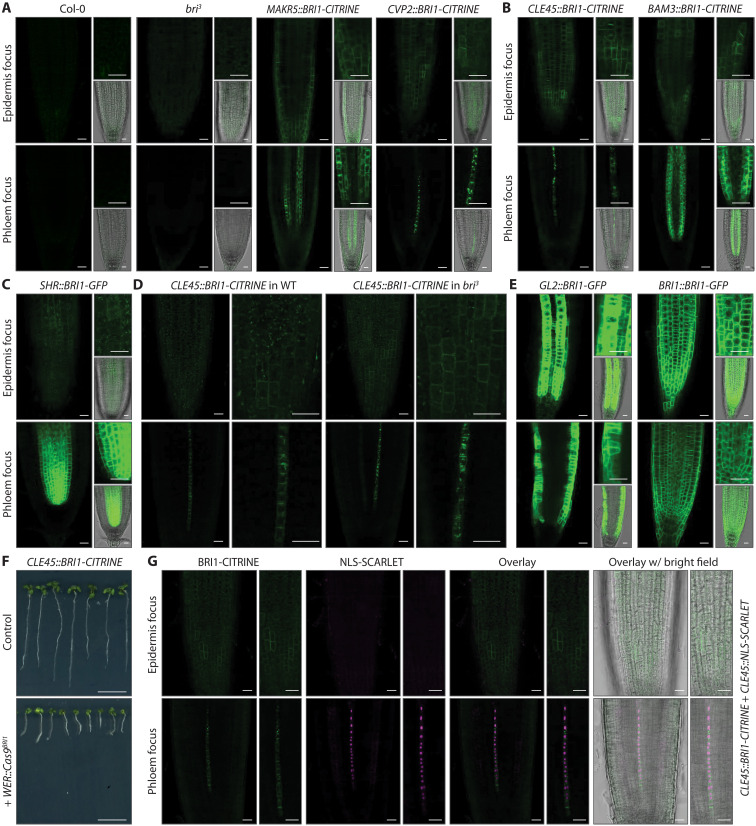
The *BRI1* gene body sequence confers low-level ubiquitous expression of trace receptor protein amounts. (**A** to **C**) Confocal microscopy images of root meristems from seedlings expressing BRI1-CITRINE or BRI1–green fluorescent protein (GFP) fusion protein under control of different tissue-specific promoters in *bri^3^* background. Note the faint plasma membrane–localized signal that is absent in Col-0 and *bri^3^* controls. (**D**) Comparison of phloem sieve element–specific *CLE45* promoter-driven BRI1-CITRINE signal in morphologically wild-type (WT) (*bri1* +/− *brl1* +/− *brl3* +/−) and *bri^3^* background. (**E**) Confocal microscopy images of root meristems from seedlings expressing BRI1-GFP fusion protein under control of the atrichoblast-specific *GL2* or the native *BRI1* promoter in *bri^3^* background. (**F**) Representative 8-day-old *bri^3^* seedlings complemented with a *CLE45::BRI1-CITRINE* transgene (top) and the same line combined with tissue-specific CRISPR-Cas9 *BRI1* knockout using the *WER* promoter (bottom). (**G**) Confocal microscopy images of a root meristem expressing both BRI1-CITRINE fusion protein (green fluorescence) and nuclear-localized NLS-SCARLET protein (magenta fluorescence) under control of the *CLE45* promoter. Note the exclusively phloem sieve element–specific NLS-SCARLET signal. Scale bars, 20 μm (microscopy images) and 1 cm (seedling images).

Considering the importance of epidermal *BRI1* expression in the *MAKR5::BRI1-CITRINE* line, we next monitored various other *bri^3^* rescue lines. In the *CVP2::BRI1-CITRINE* lines, epidermal signal was difficult to detect even if BRI1-CITRINE fluorescence in the protophloem was strong. However, when imaged without counterstaining and depending on the confocal microscopy instrument, faint plasma membrane–localized signal that was absent from background controls could be seen (Fig. 3A). Such signal was more readily detected in *CLE45::BRI1-CITRINE* and *BAM3::BRI1-CITRINE* seedlings (Fig. 3B) and was also evident in *SHR::BRI1-GFP* seedlings (Fig. 3C), indicating that it did not depend on the fluorophore. Consistently, it was also observed in newly generated *SHR::BRI1-CITRINE* seedlings (fig. S2G). Moreover, the epidermal signal did not depend on the genetic background (Fig. 3D and fig. S3A). Introduction of the *WER::Cas9^BRI1^* construct into *CLE45::BRI1-CITRINE* seedlings again triggered reversion to a *bri^3^* phenotype despite continued signal in the phloem (Fig. 3F). These experiments corroborated that the faint epidermal expression was essential for mutant complementation, although it was considerably weaker than the epidermal BRI1–green fluorescent protein (GFP) signal observed upon expression with the atrichoblast-specific *GLABRA 2* (*GL2*) promoter or the native *BRI1* promoter (Fig. 3E and fig. S1). To further pinpoint the origin of the epidermal signal, we monitored nuclear-localized NLS-SCARLET fusion protein expressed under control of the *CLE45* promoter. NLS-SCARLET was exclusively detected in developing protophloem sieve elements, both when imaged alone in wild-type or *bri^3^* background (fig. S3, B and C), and in *CLE45::BRI1-CITRINE* background (Fig. 3G). These findings suggest that the observed epidermal BRI1-CITRINE signal was not due to the promoters, the fluorophores, or vector-related regulatory elements in the T-DNA.

### Ectopic *BRI1* transgene expression is not a result of brassinosteroid feedback regulation

Matching their capacity to rescue *bri^3^*, faint epidermal signal was also detected in *CLE45::BRL1-CITRINE* and *CLE45::BRL3-CITRINE* plants but not in *CLE45::BRL2-CITRINE* plants (fig. S4, A to C). This raised the question whether the ectopic signal may result from autoregulatory feedback, because *bri^3^* rescue by BRI1 depends on brassinosteroid biosynthesis (*26*). To test this idea, we first treated *BRI1-CITRINE* lines with the brassinosteroid biosynthesis inhibitor brassinazole. This abolished *bri^3^* rescue although the ectopic BRI1-CITRINE expression persisted (fig. S4, D and E). Oppositely, brassinolide treatment did not affect the epidermal signal (fig. S4F). We also found a hypomorphic mutation (D48del) in the brassinosteroid biosynthetic enzyme DWARF1 (DWF1) (*37*) that reverts the *bri^3^* rescue in *CVP2::BRI1-CITRINE* background but can be restored by brassinolide application (fig. S4G). Epidermal BRI1-CITRINE signal in this line also did not respond to external supply of brassinolide (fig. S4H). Moreover, *BRI1* control constructs with a point mutation that abolishes BRI1 kinase activity (E1078K; *CLE45::BRI1^KDD^-CITRINE* and *CVP2::BRI1^KDD^-CITRINE*) (*38*) could not complement *bri^3^* and still displayed epidermal signal (fig. S5, A to D). Together, these experiments verify that the ectopic transgene expression was not a result of restored brassinosteroid perception itself.

### Gene body–intrinsic DNA sequences drive trace ubiquitous BRI1 gene expression

**Fig. 4.**
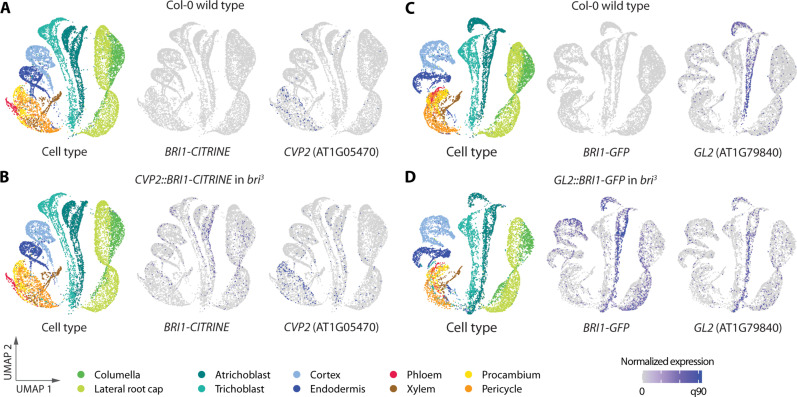
Below detection threshold levels of brassinosteroid receptor are ubiquitously expressed in brassinosteroid-blind mutants complemented with tissue-specific promoters. (**A** to **D**) Uniform Manifold Approximation and Projection (UMAP) representation of scRNA-seq data obtained from Col-0 wild-type or *bri^3^* mutant root tips (partially) complemented with BRI1-CITRINE or BRI1-GFP fusion protein expressed with either the *CVP2* (A and B) or the *GL2* promoters (C and D). Left: Cell type overviews. Dots represent cells. Middle and right: Blue dots represent cells with target gene expression. Note the difference between detected transgene transcripts and endogenous wild-type references.

In parallel, we tested whether BRI1 kinase activity is not only necessary but also sufficient to confer *bri^3^* rescue. To this end, we introduced a constitutively active chimeric receptor composed of the BAK1-INTERACTING RECEPTOR-LIKE KINASE 3 (BIR3) extracellular domain and the intracellular BRI1 kinase domain (BIR3^EXT^BRI1^INT^) (*39*) into the *bri^3^* background. The BIR3^EX^BRI1^INT^ chimera can trigger strong gain-of-function phenotypes that mimic brassinosteroid pathway hyperactivity (*39*). Expression of a BIR3^EXT^BRI1^INT^-CITRINE fusion under control of the *CLE45* promoter produced a range of phenotypes that were apparently related to transgene dosage and expression. While we noticed the described gain-of-function phenotype of short, twisted roots (fig. S5A), largely rescued yet still twisting roots were also observed (fig. S5E). However, in all seedlings, BIR3^EXT^BRI1^INT^-CITRINE fluorescence was observed throughout the root tissues (fig. S5, F and G). We also examined another chimeric receptor gene, composed of the coding regions of the BRI1 extracellular domain and the intracellular domain of the CLE peptide receptor BAM3 (BRI1^EXT^BAM3^INT^) (*34*). The *CLE45::BRI1^EXT^BAM3^INT^-CITRINE* construct could not rescue *bri^3^* plants and also did not display ectopic BRI1^EXT^BAM3^INT^-CITRINE signal (fig. S5, H and I). These experiments thus suggested not only that a constitutively activated BRI1 kinase domain can rescue *bri^3^* but also that the *BRI1* region encoding the intracellular domain confers ectopic expression in tissues other than the epidermis. To verify this notion, we reevaluated the *CVP2::BRI1-CITRINE* reference line by scRNA-seq compared to wild-type. Interrogation of these data with a dedicated *BRI1-CITRINE* reference sequence revealed low yet widespread expression of the *BRI1-CITRINE* transcript, contrasting with the more restricted expression of *CVP2* (Fig. 4, A and B, and data S1). Moreover, reanalysis of scRNA-seq data from a *bri^3^* line expressing a BRI1-GFP fusion protein under control of the *GL2* promoter (*25*) yielded a similar result with a *BRI1-GFP* reference. That is, besides in atrichoblasts as expected, *BRI1-GFP* expression was also observed in all other tissues (Fig. 4, C and D, and data S1).

**Fig. 5.**
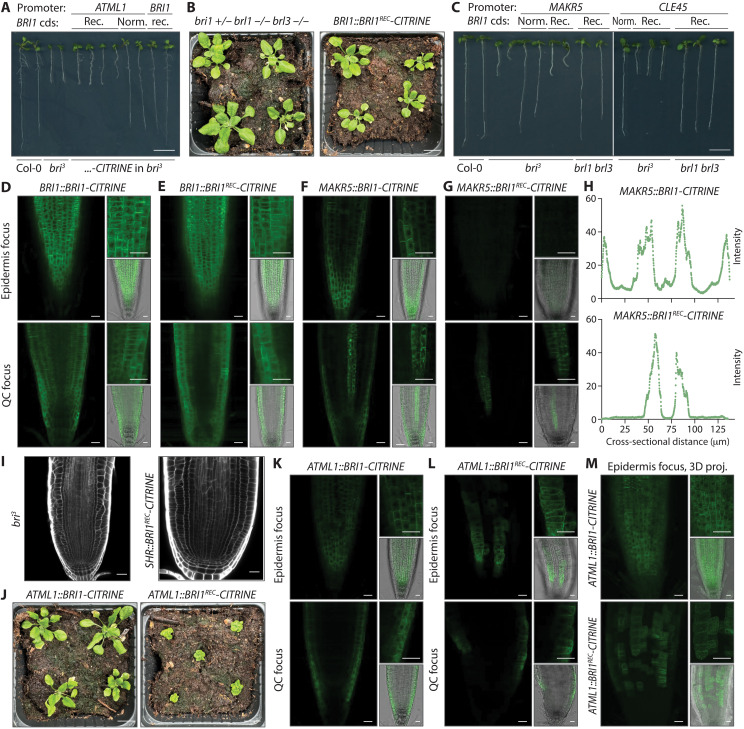
Ubiquitously expressed trace amounts of brassinosteroid receptor are necessary and sufficient to complement brassinosteroid-blind mutants. (**A**) Representative 8-day-old seedlings carrying *BRI1-CITRINE* transgenes made with the genuine (Norm.) *BRI1* coding sequence (cds) or a recoded version (Rec.; ~25% of nucleotides exchanged; *BRI1::BRI1^REC^-CITRINE*), compared to wild-type and *bri^3^* controls. Note the difference in seedlings expressing either version under the epidermis-specific *ATML1* promoter. (**B**) Three-week-old *bri^3^* mutants complemented by a *BRI1::BRI1^REC^-CITRINE* transgene (right) compared to morphologically wild-type *brl1 brl3* double mutants segregating from the transformed parental line (left). (**C**) Representative 8-day-old seedlings expressing BRI1-CITRINE or BRI1^REC^-CITRINE fusion protein under control of the *MAKR5* and *CLE45* promoters in *bri^3^* mutants and morphologically wild-type *brl1 brl3* double mutants, compared to Col-0 and *bri^3^* controls. (**D** and **E**) Confocal microscopy images of root meristems from seedlings expressing BRI1-CITRINE fusion proteins from the original or recoded *BRI1* cds under control of the native *BRI1* promoter. (**F** and **G**) Confocal microscopy images of root meristems from seedlings expressing BRI1-CITRINE or BRI1^REC^-CITRINE fusion protein under control of the *MAKR5* promoter in *bri^3^* mutants, illustrating the absence of epidermal BRI1^REC^-CITRINE signal. (**H**) Quantification of CITRINE fluorescence intensity (arbitrary units) across root sections running from epidermal to epidermal cell layer through the phloem poles. (**I**) Confocal microscopy images of calcofluor-stained root meristems from *bri^3^* mutants (left) and their counterparts expressing BRI1^REC^-CITRINE fusion protein under control of the *SHR* promoter (right). (**J**) Three-week-old *bri^3^* mutants carrying an *ATML1::BRI1-CITRINE* (left) or *ATML1::BRI1^REC^-CITRINE* transgene (right). Note the difference compared to the complementation obtained with a *BRI1::BRI1^REC^-CITRINE* transgene (B). (**K** to **M**) Confocal microscopy images of root meristems from the indicated genotypes. QC, quiescent center. 3D projections focused on the epidermis are shown in (M). Scale bars, 20 μm (microscopy images) and 1 cm (seedling images).

Because brassinosteroid receptor genes in Arabidopsis as well as other species typically do not contain any introns (https://phytozome-next.jgi.doe.gov) (*40*), we concluded that ectopic *BRI1* transgene expression may reflect an inherent feature of the *BRI1* coding sequence. To directly test this idea, we engineered a recoded version of *BRI1* (*BRI1^REC^*) in which 907 of the 3591 nucleotides were exchanged (fig. S6). The *BRI1^REC^* gene sequence thus produces a protein that is identical to BRI1 but uses different codons for 738 of the 1197 BRI1 amino acids. When BRI1^REC^-CITRINE fusion protein was expressed under the control of the *BRI1* promoter, it complemented the *bri^3^* mutant (Fig. 5, A, B, D, and E) like *BRI1:BRI1-CITRINE*, *BRI1:BRL1-CITRINE*, or *BRI1:BRL3-CITRINE* controls (fig. S7, A and B), confirming BRI1^REC^ functionality. However, when expressed with phloem-specific promoters, no rescue was observed (Fig. 5C and fig. S7C), although BRI1^REC^-CITRINE was readily detectable in the phloem, whereas epidermal expression was absent (Fig. 5, F and G, and fig. S7, D and E). The expression levels of BRI1^REC^-CITRINE were comparable to those of BRI1-CITRINE in the promoter expression domains. However, fluorescence intensity quantification across root sections confirmed the absence of above-background BRI1^REC^-CITRINE signal in the epidermis or other tissues, whereas BRI1-CITRINE signal was evident in the epidermis and also above-background elsewhere (Fig. 5H).

The observation that *bri^3^* rescue by *CVP2::BRI1-CITRINE* is accompanied by resistance to external application of CLE45 peptide in a dosage-dependent manner (*26*) gave us a handle to independently verify BRI1^REC^-|CITRINE functionality in the phloem. CLE45 peptide is an autocrine regulator of phloem formation and produced by the *CLE45* gene (*34*, *41*). Hyperactivation of CLE45 signaling, for instance by application of synthetic CLE45 peptide at nanomolar concentrations, suppresses phloem formation and thereby root growth (*34*). CLE45 resistance was observed whenever *BRI1* was expressed under protophloem (pole)–specific promoters (fig. S7F). Moreover, *MAKR5::BRI1-CITRINE bri^3^* seedlings that carried the *SHR::Cas9^BRI1^* construct and had lost BRI1-CITRINE signal in the phloem pole also had lost their CLE45 resistance (fig. S7G), corroborating that it is a consequence of high BRI1 activity in the phloem (*26*, *41*). Consistently, *bri^3^* or wild-type–like seedlings that expressed a *MAKR5::BRI1^REC^-CITRINE* transgene displayed CLE45 resistance (fig. S7H), again confirming that BRI1^REC^-CITRINE is functional. In summary, our data suggest that the *BRI1* gene body contains regulatory sequences that confer low-level ubiquitous gene expression throughout the root and are lost in a recoded *BRI1* gene.

### Brassinosteroid signaling in individual tissues is not sufficient for *bri*^*3*^ complementation

Whether trace amounts of BRI1 in tissues other than the epidermis and phloem vasculature are also required for comprehensive *bri^3^* rescue was difficult to determine but appeared likely. For instance, CRISPR-Cas9–mediated *BRI1* knockout in the cortex cell layer was recently shown to largely revert complementation of *bri1* single mutants (*25*). Oppositely, expression of a BRI1-GFP fusion protein with a cortex-specific (*COR*; AT1G09750) promoter complemented *bri^3^* root growth (fig. S8A), but consistently, these seedlings also showed epidermal BRI1-GFP signal (fig. S8B). To determine whether brassinosteroid signaling in any individual tissue is sufficient to rescue the root growth of *bri^3^*, we expressed the BRI1^REC^ variant with additional promoters. BRI1^REC^-CITRINE expressed under the *SHR* promoter showed no epidermal signal (fig. S8, C and D) and conferred no rescue (fig. S8E), but higher levels triggered excess formative divisions in the stele (Fig. 5I and fig. S8F), consistent with previous reports (*13*, *17*, *18*). These excess divisions were not observed when BRI1^REC^-CITRINE was expressed under the control of the other promoters, suggesting that they reflect local effects of (hyperactive) brassinosteroid signaling. Last, we also expressed BRI1^REC^-CITRINE under the control of the epidermis-specific *ARABIDOPSIS THALIANA MERISTEM LAYER 1* (*ATML1*) promoter (*42*) (fig. S1), which had been used in the original transgenic complementation of *bri1* shoot growth (*32*). However, unlike in *ATML1::BRI1-CITRINE* controls, neither shoot nor root growth defects were complemented in *ATML1::BRI1^REC^-CITRINE* lines (Fig. 5, A and J) despite epidermal BRI1^REC^-CITRINE signal (Fig. 5, K and L). Moreover, the BRI1-CITRINE signal was spread more evenly throughout the epidermis, whereas the BRI1^REC^-CITRINE signal resembled the *ATML1* expression pattern (Fig. 5M and fig. S1). Similar observations were made when BRI1^REC^-CITRINE was instead expressed under control of the *GL2* promoter (fig. S8, G to J). Together, these results indicate that expression of the brassinosteroid receptor in the *ATML1* or *GL2* expression domains of the epidermis is not by itself sufficient for *bri^3^* rescue.

## DISCUSSION

Loss-of-function genetics has established BRI1 as the dominant Arabidopsis brassinosteroid receptor because it is essential for growth (*5*, *43*), whereas minor roles or specific functions were assigned to its homologs BRL1 and BRL3 (*3*, *44*). In *bri1* single mutants, *BRL1* and *BRL3* are presumably still active in stele tissues. Rescue of the *bri^3^* phenotype by BRL1 or BRL3 expression under control of tissue-specific promoters may therefore appear counterintuitive. However, we have shown previously that the extent of *bri^3^* rescue through phloem-specific *BRI1* expression depends on expression levels and transgene copy number (*18*, *26*), and even single-locus transgene insertions are typically concatenated (*45*). Thus, our data reiterate the importance of quantitative brassinosteroid signaling and indicate that *BRL1* and *BRL3* expression is normally below the threshold to compensate for the absence of *BRI1*. Moreover, differences in rescue efficiency may also reflect the local impact of brassinosteroid signaling on brassinosteroid homeostasis. For example, the rescue observed with phloem pole–specific promoters may be enhanced by the regulation of genes encoding rate-limiting brassinosteroid biosynthesis enzymes, which are expressed in the vascular cylinder (*19*, *28*).

**Fig. 6.**
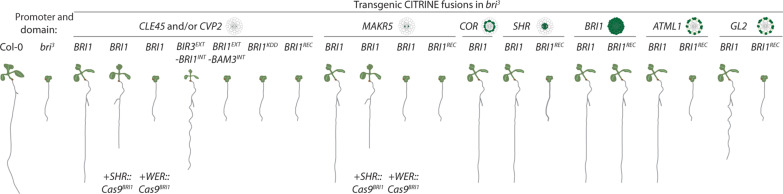
Schematic overview. Key BRI1-related transgenic lines produced in this study and their representative phenotypes.

A central deduction from our combination of promoters and *BRI1* coding sequences in genetic analyses (Fig. 6) is that DNA sequences in the coding region of brassinosteroid receptor genes contribute to their expression pattern and confer low expression throughout root tissues. This is most evident in the ectopic receptor signal observed with stronger phloem-specific promoters, which also suggests a synergism between regulatory sequences in the promoter and the gene body. The contribution of the transcript region to gene expression pattern has so far only been observed rarely (*46*–*48*). A comprehensive comparison of promoter-driven reporter gene expression patterns with scRNA-seq data could reveal how common this phenomenon is. Our observations with chimeric receptor genes and a recoded *BRI1* gene suggest that the region encoding the intracellular domain is necessary for the ubiquitous trace *BRI1* expression. Future analyses of more refined chimera may enable identification of the responsible sequence tracts, which could be aided by exploring possibly epigenetic regulation as well as analyses of *BRI1* homologs from other plants. Moreover, it is conceivable that conservation of trace ubiquitous expression may be a driving force in brassinosteroid receptor gene evolution and underlie the observation that they rarely contain introns.

Collectively, our confocal microscopy, scRNA-seq, and genetic data suggest that *BRI1* transgenes are weakly expressed throughout root tissues and that trace amounts of BRI1 are frequently sufficient to sustain root growth in the *bri^3^* background. This is unexpected given that the ectopic BRI1-CITRINE/GFP signal can be below the detection threshold of contemporary confocal microscopes. In general, it was only clearly evident as above-background fluorescence plasma membrane–localized signal in the absence of any counterstaining. The necessity of the ectopic epidermal expression for *bri^3^* complementation reiterates the importance of the epidermis in restricting organ growth (*32*). Yet, our results also suggest that a threshold level of brassinosteroid perception across multiple, if not all, tissues is necessary for comprehensive *bri^3^* rescue. The results may also mean that a lower level of brassinosteroid signaling across tissues is preferable over a strong imbalance between inner and outer cell layers, which may sometimes prevent phenotypic recovery as previously suggested (*17*, *27*). Conversely, restricted expression of the brassinosteroid receptor in individual tissues is not sufficient to normalize the *bri^3^* mutant phenotype. The most parsimonious interpretation of our data is therefore that brassinosteroid receptors largely act in a cell-autonomous manner. A cell-autonomous action of brassinosteroid perception does not exclude that brassinosteroid itself could act non–cell autonomously. Brassinosteroids are polyhydroxylated steroids that cannot easily diffuse across membranes (*14*, *28*). The recently characterized differential biosynthesis and targeted distribution of active brassinosteroids and their precursors (*28*, *49*, *50*) therefore likely have a pivotal role in shaping local differences in brassinosteroid action and brassinosteroid-dependent growth coordination (*13*, *17*, *18*, *27*, *31*). Our results thus also suggest that the *BRI1* gene safeguards the capacity of individual cells to respond to differential brassinosteroid cues and moreover that, in general, local levels of active brassinosteroids rather than BRI1 receptor levels are limiting brassinosteroid response.

## MATERIALS AND METHODS

### Plant materials and growth conditions

All the lines used in this work were in the Arabidopsis wild-type accession Columbia-0 (Col-0) background. The *bri1-116 brl1 brl3* triple mutant (*bri^3^*) and the *CVP2::BRI1-CITRINE bri^3^*, *BAM3::BRI1-CITRINE bri^3^*, *MAKR5::BRI1-CITRINE bri^3^*, *SHR::BRI1-GFP bri^3^*, *GL2::BRI1-GFP bri^3^*, and *BRI1::BRI1-GFP bri^3^* transgenic lines were described previously (*13*, *26*, *27*). For in vitro culture, seeds were surface sterilized and then stratified at 4°C for 2 to 3 days. Seeds were placed on plates containing one-half Murashige and Skoog medium (including vitamins and MES buffer; Duchefa, M0255) supplemented with 1% agar and 0.3% sucrose. The pH was adjusted to 5.7. Seedlings were grown vertically in a growth chamber under continuous white light of ~120 μmol m^−2^ s^−1^ intensity at 22°C. CLE peptides were obtained from a commercial supplier (Genscript; synthesized at >80% purity), diluted in water, and added to the medium at a final concentration of 15 nM. For root growth assays, brassinolide (Sigma-Aldrich, product no. SML0094) and brassinazole (Tokyo Chemical Industry, product no. B2829) were applied at the indicated final concentrations. For root growth measurements, plates scanned with a high-resolution flatbed scanner were analyzed using the Simple Neurite Trace plug-in for Fiji software. All the experiments were repeated at least once.

### Transgenic brassinosteroid receptor and reporter lines

For transgene constructs, attB-flanked coding sequences were synthetized (*BRI1^REC^*, *BRI1^KDD^*, and *BRI1^EXT^BAM3^INT^*) or amplified (*BIR3^EXT^-BRI1^INT^*, *BRI1*, *BRL1*, *BRL2*, and *BRL3*) by polymerase chain reaction and recombined into the pDONR221 vector (Invitrogen) to produce the pEN-L1-CDS-L2 clones. The *BRI1^REC^* sequence was generated by the GeneOptimizer recoding algorithm (Thermo Fisher Scientific) using the normal *BRI1* sequence as a template (see the Supplementary Materials, data S2 for sequences). Protein fusions under the control of different promoters were generated by recombining pEN-L4-promoter-R1, pEN-L1-CDS-L2, and pEN-R2-CITRINE-L3 plasmids into a destination vector by Multisite Gateway LR reaction (Thermo Fisher Scientific). The *BRI1*, *CLE45*, *COR*, *CVP2*, *ATML1*, *GL2*, and *SHR* promoters have been previously described (*13*, *25*, *27*, *32*, *42*, *51*). The destination vectors used were pFR7m34GW (for *CLE45::NLS-SCARLET* and *BRI1^REC^*constructs), pK8m34GW-FAST (for *COR::BRI1-GFP*) and pH7m34GW (for all other constructs). All constructs were verified by Sanger sequencing and then introduced into *Agrobacterium tumefaciens* strain GV3101 with the pMP90 helper plasmid. To obtain the different transgenes in *bri^3^* background, homozygous *brl1 brl3* double-mutant plants that were heterozygous for the *bri1-116* allele were transformed by the floral dip method. Transgenics in homozygous *bri^3^* background were selected by genotyping as described (*26*, *27*). The *COR::BRI1-GFP* construct was transformed into a *WOX5::erGFP bri^3^* background, the *CLE45::NLS-SCARLET* construct also into Col-0 and *bri^3^* background. For each construct-background combination, several independent transgenic lines were obtained. Observations were typically confirmed in detail in two or more independent transgenic lines.

### *BRI1* transgene knockout by tissue-specific CRISPR-Cas9

The *pEN-R2-gRNA_BRI1-3-gRNA_BRI1-2-L3* vector, which contains two gRNAs targeting *BRI1*, as well as the *WER::Cas9^BRI1^* expression construct have been previously described (*25*). The *SHR::Cas9^BRI1^* expression clone was generated by combining the pDONRL4-L1r plasmid carrying the *SHR* promoter, the *pEN-R2-gRNA_BRI1-3-gRNA_BRI1-2-L3* plasmid, and the destination vector pK8m34GW-FAST in a MultiSite Gateway LR reaction. *pCVP2::BRI1-CIT bri^3^*, *pCLE45::BRI1-CIT bri^3^*, and *pMAKR5::BRI1-CIT bri^3^* plants were transformed with both *WER::Cas9^BRI1^* and *SHR::Cas9^BRI1^* expression constructs by the floral dip method. Transgenic T2 generation plants were selected on the basis of the presence of GFP signal in the seed coat, and the efficiency of the tissue-specific CRISPR was confirmed by confocal microscopy.

### *DWF1* knockout by CRISPR-Cas9

Three gRNAs (5′-ACT CTG ACC ACA TGT CCC CG-3′, 5′-ATC TTT ACT ACG CAA TCC CG-3′, and 5′-CTA CTT CCT CAT CTA CCT CG-3′) were designed to target the first exon of *DWF1* (AT3G19820) in the *pCVP2::BRI1-CITRINE bri^3^* line. T1 generation plants were selected on the basis of FASTRED, and the mutations were confirmed by genotyping and sequencing. The following primers were used: *2F*, 5′-TGG TTT GAT GCA GTG A-3′; *DWF1 crispr geno 2R*, 5′-CAC GGC TTG AAC CAC-3′. Different alleles were found, and the hypomorhpic *dwf1^del48^* allele was chosen for further experiments because it is able to produce seeds.

### Confocal microscopy

Live confocal microscopy was performed in most cases. For detection of fluorescent proteins, the following emission-excitation wavelengths in a Leica Stellaris 5 instrument were used: excitation 488 or 514 nm/emission 493 to 565 nm (GFP/CITRINE) and excitation 561 nm/emission 566 to 734 nm (NLS-SCARLET). Formative cell divisions were analyzed in 7-day-old roots fixed with 4% paraformaldehyde in phosphate-buffered saline (PBS) buffer for 30 min and cleared with ClearSee solution for 5 days. Cleared roots were stained with 0.1% Calcofluor White (CAS-No: 4193-55-9; Sigma-Aldrich) in ClearSee solution and washed two times with PBS buffer (10 to 15 min each). Samples were imaged on a Leica Stellaris 5 confocal microscope with 20× and 63× objectives, using the 405-nm laser for calcofluor excitation. For image analyses, Fiji software was used.

### 10x Genomics scRNA-seq of Arabidopsis root protoplasts

scRNA-seq analysis of *CVP2::BRI1-CITRINE* in *bri^3^* was performed in a side-by-side experiment along with published samples for wild-type, *bri^3^*, and *GL2::BRI1-GFP* in *bri^3^* as previously described (*25*). Plants were grown vertically in a growth chamber set to 22°C, 16-hour light/8-hour dark for 7 days on 1/2 Linsmaier and Skoog (LSP03-1LT, Caisson Labs; pH 5.7) 1% sucrose media with 100-μm nylon mesh (Nitex 03-100/44). Root tips were harvested from 1000 to 3000 roots per sample by cutting ~0.5 cm from the tip with a razor blade. Excised roots were placed into a 35-mm petri dish containing a 70-μm cell strainer and 4.5 ml of enzyme solution [1.5% (w/v) cellulase (ONOZUKA R-10, GoldBio), 0.1% Pectolyase (Sigma-Aldrich, P3026), 0.4 M mannitol, 20 mM MES (pH 5.7), 20 mM KCl, 10 mM CaCl_2_, 0.1% bovine serum albumin, and 0.000194% (v/v) β-mercaptoethanol]. The digestion was incubated on an 85-rpm shaker at 25°C for 1 hour with additional stirring every 15 to 20 min. The resulting cell solution was filtered twice through 40-μm cell strainers and centrifuged for 5 min at 500*g* in a swinging bucket rotor. The pellet was washed with 2 ml of washing solution [0.4 M mannitol, 20 mM MES (pH 5.7), 20 mM KCl, 10 mM CaCl_2_, 0.1% bovine serum albumin, and 0.000194% (v/v) β-mercaptoethanol] and centrifuged again at 500*g* for 3 min, and the pellet was resuspended in washing solution at a concentration of ~2000 cells/μl. We loaded 16,000 cells, with the aim to capture 10,000 cells per sample with the 10x Genomics Chromium 3' Gene expression v3.1 kits. Cell barcoding and library construction were performed following the manufacturer’s instructions. cDNA and final library quality were verified using a Bioanalyzer High Sensitivity DNA Chip (Agilent) and sequenced on an Illumina NovaSeq 6000 instrument.

### scRNA-seq data processing and analysis

scRNA-seq analysis was carried out as described (*25*), except that the genome sequences were modified to analyze the *BRI1* transgenes. Sequencing reads were demultiplexed from Illumina BCL files to produce FASTQ files for each sample using CellRanger mkfastq (v3.1.0, 10x Genomics). We then created two separate custom reference files using the Arabidopsis TAIR10 reference genome. The first contained the *BRI1-CITRINE* transgene sequence, which was used to analyze wild type and *CVP2::BRI1-CITRINE* in *bri^3^*, while the second contained *BRI1-GFP* and was used to analyze wild type and *GL2::BRI1-GFP* in *bri^3^*. Reads were then aligned against the custom reference to generate a gene-by-cell matrix using the scKB script (https://github.com/ohlerlab/scKB), which incorporates kallisto and bustools (*52*, *53*). Quality filtering of cells was performed using the R package COPILOT (Cell preprOcessing PIpeline kaLlistO busTools) (*54*), which uses a nonarbitrary scheme to remove empty droplets and dying or low-quality cells. One iteration of COPILOT filtering adequately separated high-quality cells from the background in these samples based on an examination of barcode rank plots. The resulting high-quality cells were further filtered to remove outliers based on the top 1% of cells in terms of Unique Molecular Identifier (UMI) counts, and putative doublets were removed with DoubletFinder (*55*), incorporating the estimated doublet rate from the 10x Genomics Chromium Single Cell 3' Reagent Kit user guide. In total, we identified 20,957 high-quality cells from two biological replicates of *CVP2::BRI1-CITRINE* in *bri^3^*.

Normalization, annotation, and integration of scRNA-seq datasets were carried out using Seurat. Data were normalized using SCTransform (*56*). All genes except those from mitochondria, chloroplasts, or those affected by protoplasting (*57*, *58*) (absolute log_2_ fold change ≥ 2) were retained for analysis. Cell type and developmental stage labels from the wild-type atlas (*25*, *58*) were transferred to each sample via label transfer in Seurat (*59*, *60*). We integrated the samples from each custom reference using the Seurat integration pipeline. A sample from the atlas with the highest number of detected genes (sc_12) (*58*) and two previously described samples (dc_1 and dc_2) (*57*) were included in the integration to facilitate comparable visualizations but were excluded from any downstream analysis. Principal components analysis (PCA) was performed by calculating 50 principal components using the RunPCA function (with approx = FALSE). Uniform Manifold Approximation and Projection (UMAP) nonlinear dimensionality reduction was next calculated via the RunUMAP function using all 50 principal components with parameters n_neighbors = 30, min_dist = 0.3, umap.method = “umap-learn”, and metric = “correlation” using the “integrated” assay. These processing steps have been previously described (*25*, *58*) and are documented in Jupyter notebooks as part of the COPILOT workflow. Gene expression patterns were examined by plotting the normalized expression values produced by the SCTransform function, with the 90th quantile of expression as the maximum cutoff. To quantify *BRI1* transgene levels in different cell types, we used muscat (multi-sample multi-group scRNA-seq analysis tools) (*61*) to aggregate cell level counts for each cell type on a per-sample basis. Raw counts were summed using the aggregateData function, and differential expression testing was performed using edgeR (*62*) incorporated in the pbDS function of muscat. A gene was considered differentially expressed in a given cell type if the false discovery rate–adjusted *P* value was ≤0.05, absolute fold change was ≥ 1.5, and detection frequency was ≥ 5% in one of the genotypes. Tables were exported from muscat with counts per million normalized expression values for each cell type/sample combination.

### Statistical analysis

Data were analyzed using GraphPad Prism software version 10.2.1. Robust regression and outlier removal analyses were performed on root measurements to detect (rare) outliers, which were removed. Specific statistical tests used are indicated in the figure legends and were always two-sided.
