# Supplementary material for: The brassinosteroid receptor gene BRI1 safeguards cell-autonomous brassinosteroid signaling across tissues
Source: Sci Adv. 2024 Sep 25;10(39):eadq3352. doi: 10.1126/sciadv.adq3352 (PMC11423886; doi:10.1126/sciadv.adq3352)
Supplement: Supplementary file 1 — Figs. S1 to S8 Legends for data S1 and S2 [file sciadv.adq3352_sm.pdf]

Science Advances
AAAS

Supplementary Materials for

The brassinosteroid receptor gene *BRI1* safeguards cell-autonomous brassinosteroid signaling across tissues

Noel Blanco-Touriñán *et al.*

Corresponding author: Christian S. Hardtke, christian.hardtke@unil.ch

Sci. Adv. **10**, eadq3352 (2024)
DOI: 10.1126/sciadv.adq3352

The PDF file includes:

Figs. S1 to S8
Legends for data S1 and S2

Other Supplementary Material for this manuscript includes the following:

Data S1 and S2

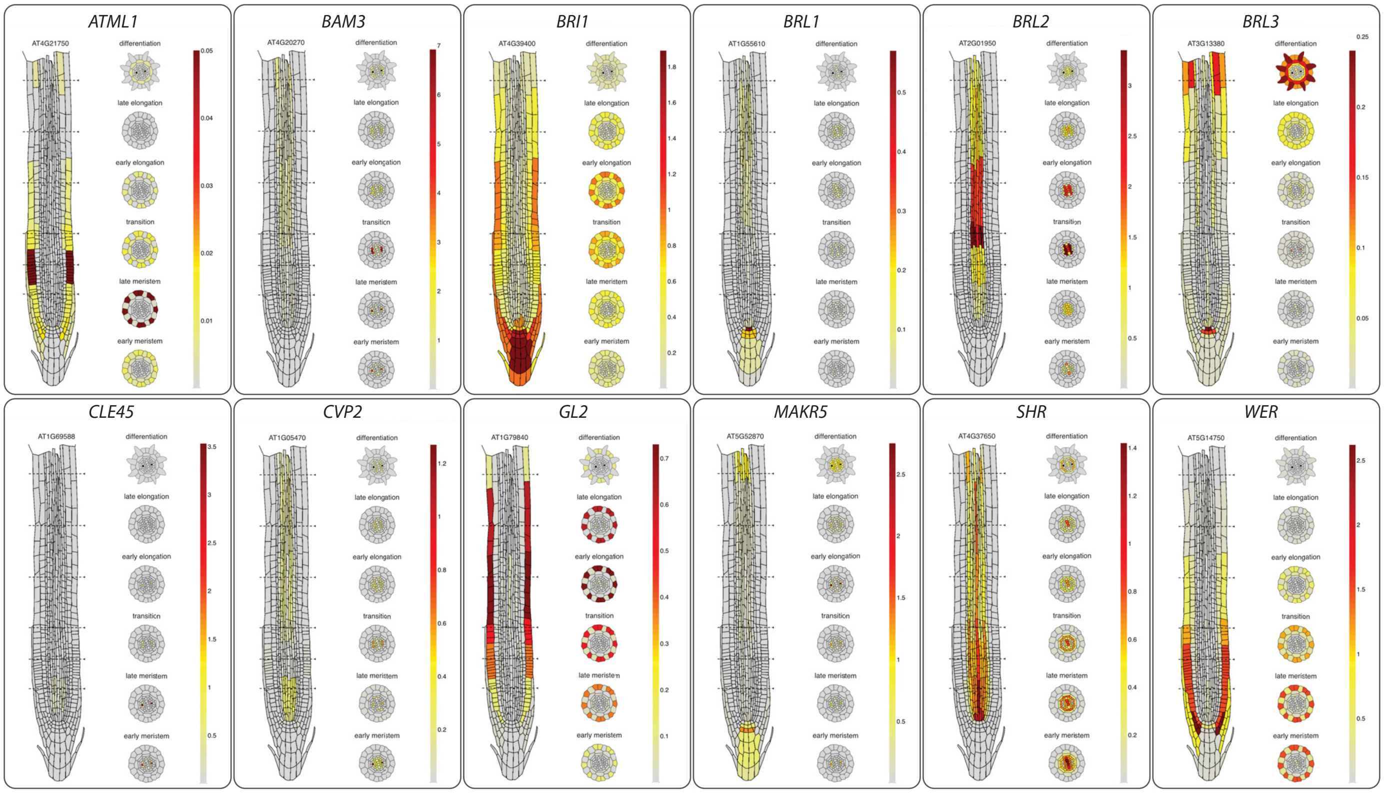

fig. S1. Expression patterns of genes investigated in this study. Schematic representation of root tip expression patterns of indicated genes, obtained from aggregation of multiple independent single cell mRNA analyses of Arabidopsis Col-0 wildtype roots (https://rootcellatlas.org). Note the differences in expression level scales.

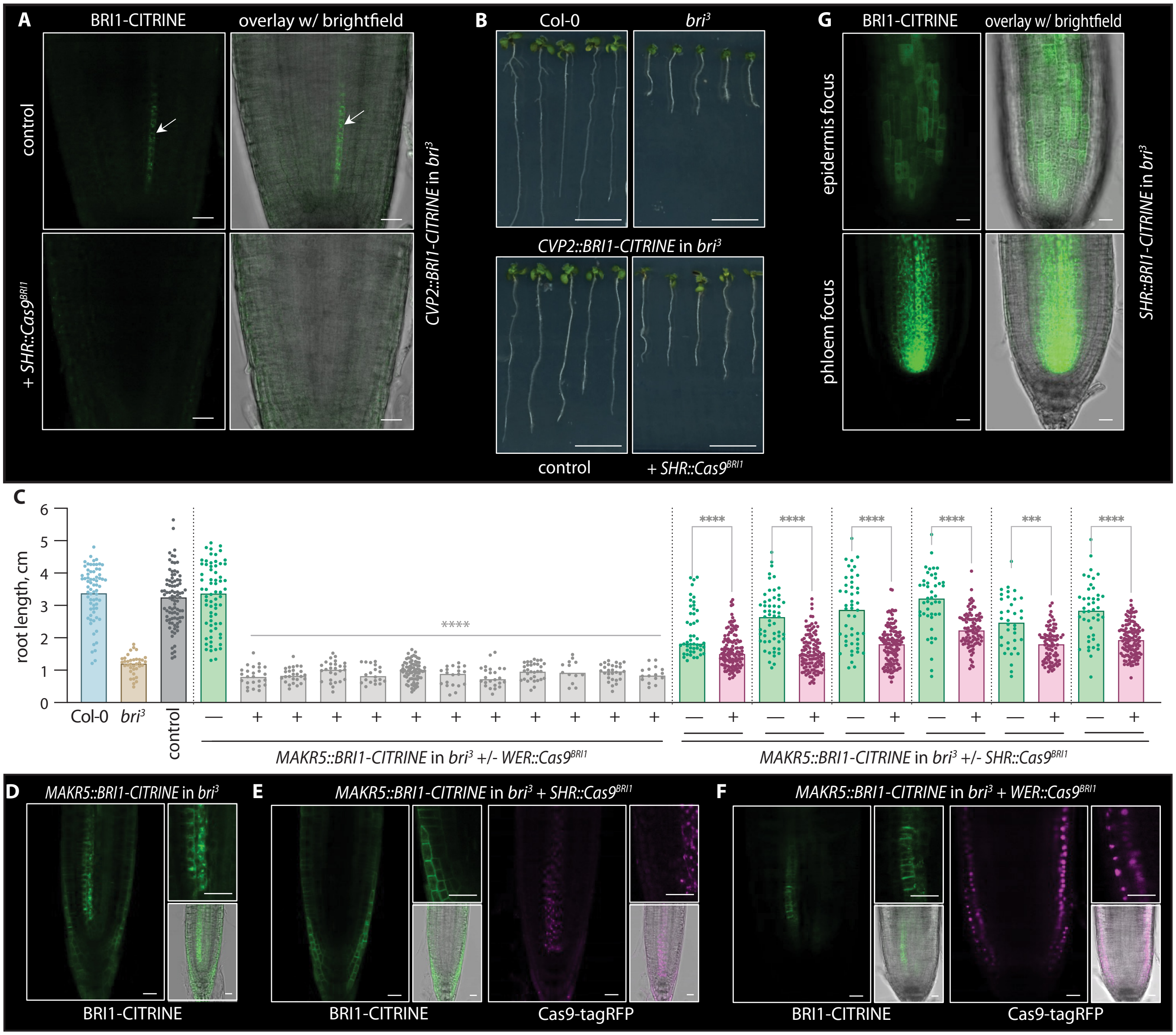

fig. S2. Trace amounts of epidermal BRI1 expression are required for *bri³* rescue. (**A**) Top: Confocal microscopy images of a root meristem from a *bri³* seedling complemented with a BRI1-CITRINE fusion protein (green fluorescence) expressed under control of the phloem sieve element-specific *CVP2* promoter, imaged without any counterstaining. Bottom: CRISPR/Cas9 knockout of the *BRI1* transgene using the stele-specific *SHR* promoter leads to disappearance of BRI1-CITRINE signal. (**B**) Representative 8-day-old *bri³* seedlings complemented with a *CVP2::BRI1-CITRINE* transgene (bottom left) and the same line combined with stele-specific CRISPR/Cas9 *BRI1* knockout (bottom right) as compared to controls (top). (**C**) Root growth quantification for 8-d-old seedlings of the indicated genotypes. A *bri³* line complemented with *BRI1-CITRINE* expressed under control of the phloem pole-specific *MAKR5* promoter (control) was combined with tissue-specific CRISPR/Cas9 *BRI1* knockout using either the stele-specific *SHR* or the epidermis-specific *WER* promoter, several independent lines are shown. Statistically significant differences (asterisk) between seedlings carrying the CRISPR/Cas9 construct and their segregating non-transgenic siblings were determined by ordinary one-way ANOVA, $p<0.0001$. (**D-F**) Confocal microscopy images of root meristems from *bri³* seedlings complemented with *BRI1-CITRINE* expressed under control of the *MAKR5* promoter (D) and combined with tissue-specific CRISPR/Cas9 *BRI1* knockout using the *SHR* (E) or *WER* (F) promoters, showing both BRI1-CITRINE and Cas9-tagRFP signals. (**G**) Confocal microscopy images of a root meristem from a *bri³* seedling expressing BRI1-CITRINE fusion protein under control of the *SHR* promoter. Size bars are 20µm in microscopy images and 1cm in seedling images.

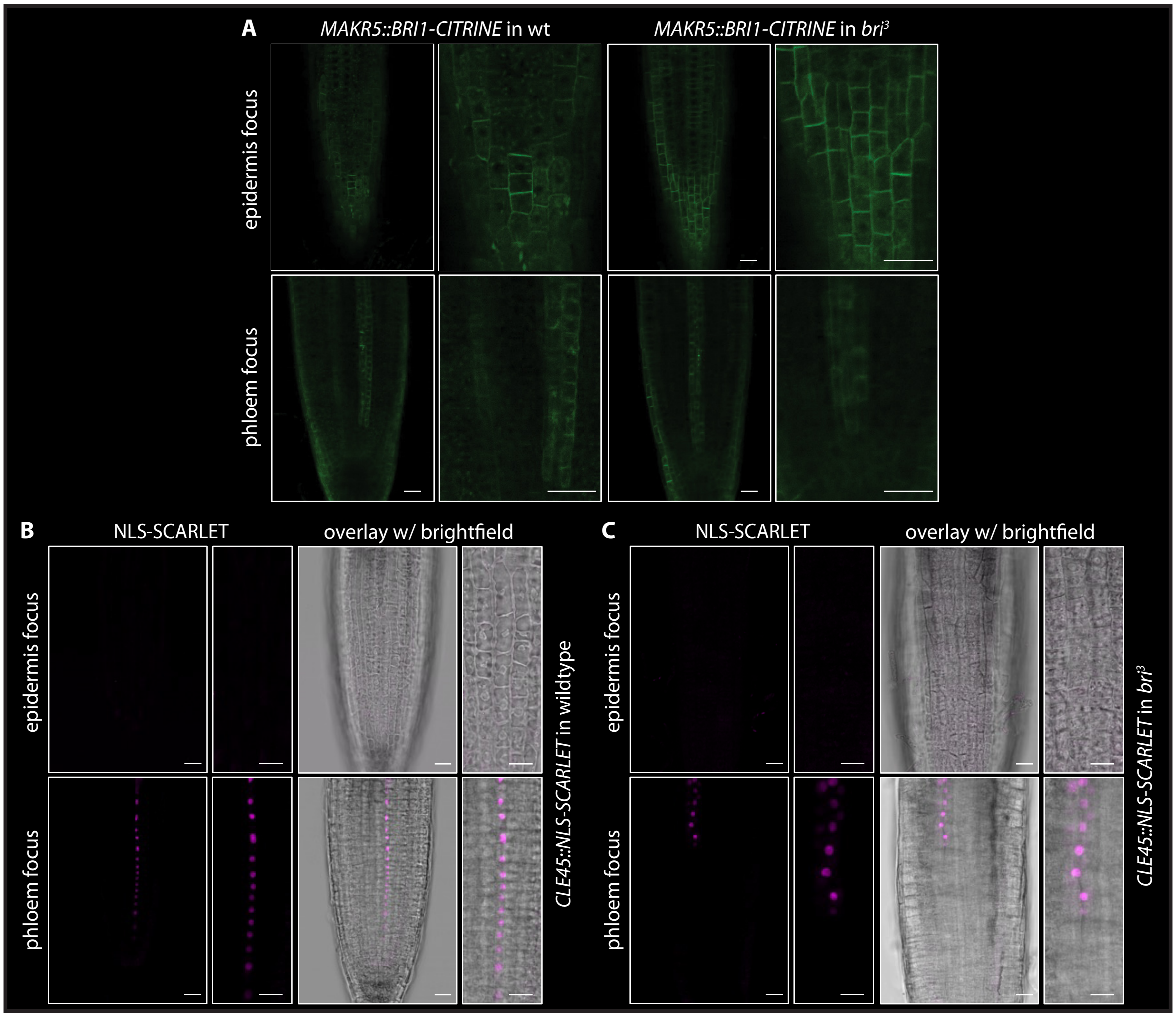

fig. S3. Trace epidermal BRI1 expression is independent of the promoter. (**A**) Comparison of phloem pole-specific *MAKR5* promoter-driven BRI1-CITRINE signal in morphologically wildtype (*bri1* +/- *brl1* +/- *brl3* +/-) and *bri*^{3} background. (**B-C**) Confocal microscopy of an NLS-SCARLET fusion protein expressed under control of the phloem sieve element-specific *CLE45* promoter in Col-0 wildtype and *bri*^{3} background. Size bars are 20μm.

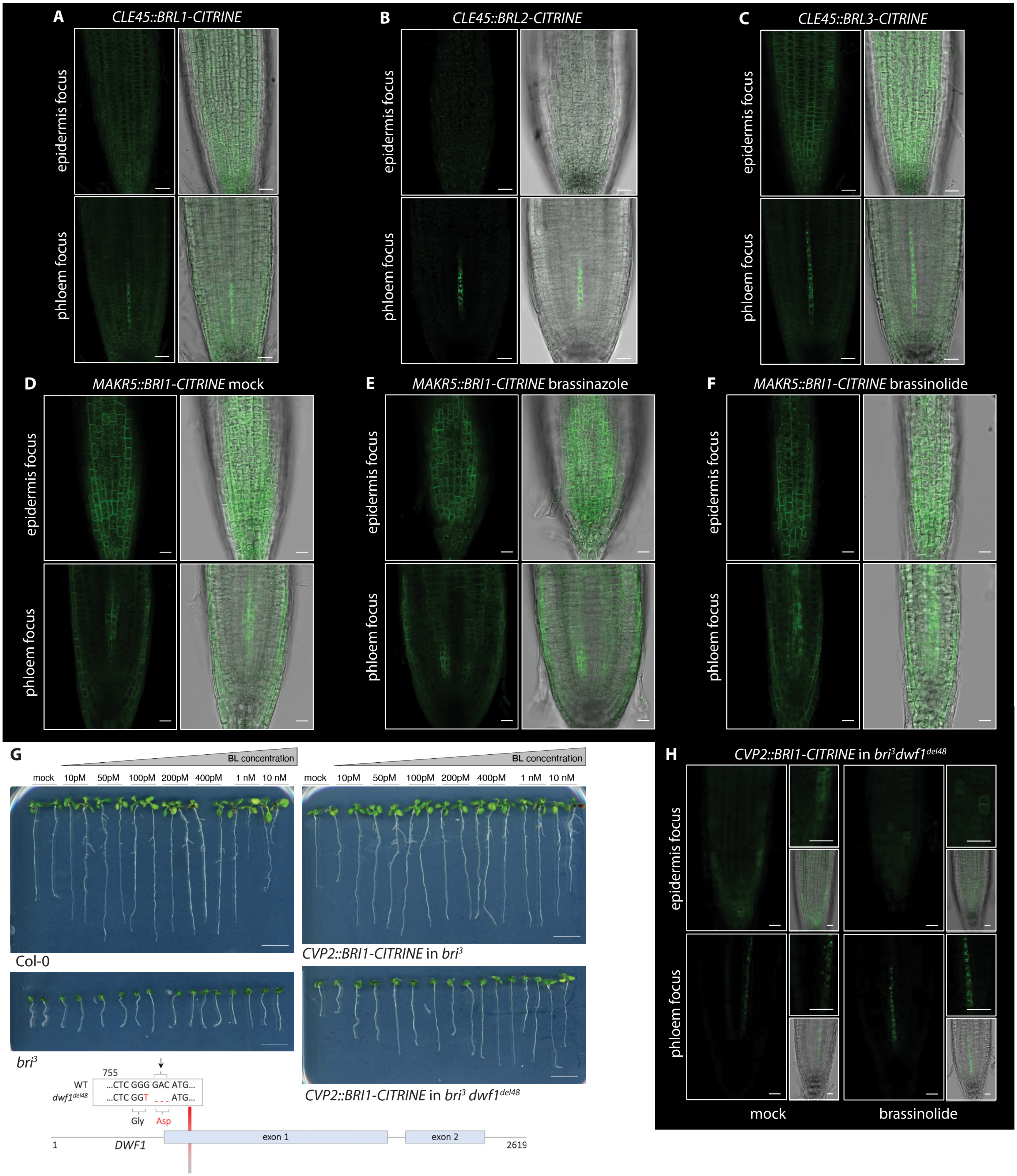

fig. S4. Trace epidermal BRI1 expression does not result from autoregulatory feedback. (**A-C**) Confocal microscopy images of root meristems from *bri*^{3} seedlings expressing CITRINE fusion proteins (green fluorescence) of the indicated BRI1 homologs under control of the phloem sieve element-specific *CLE45* promoter. (**D-F**) Confocal microscopy images of root meristems from *bri*^{3} seedlings expressing BRI1-CITRINE fusion protein under control of the phloem pole-specific *MAKR5* promoter, treated with mock, 1mM brassinazole, or 10nM brassinolide. (**G**) Representative 9-day-old *bri*^{3} seedlings complemented with a *CVP2::BRI1-CITRINE* transgene and the same line combined with a hypomorphic mutation in the brassinosteroid biosynthesis gene *DWARF1* (*dwf1*^{del48}; see schematic), compared to controls and treated with increasing levels of brassinolide. (**H**) Confocal microscopy images of a root meristem from *bri*^{3} *dwf1*^{del48} quadruple mutant seedlings carrying a *CVP2::BRI1-CITRINE* transgene, treated with mock or 100nM brassinolide. Size bars are 20µm in microscopy images and 1cm in seedling images.

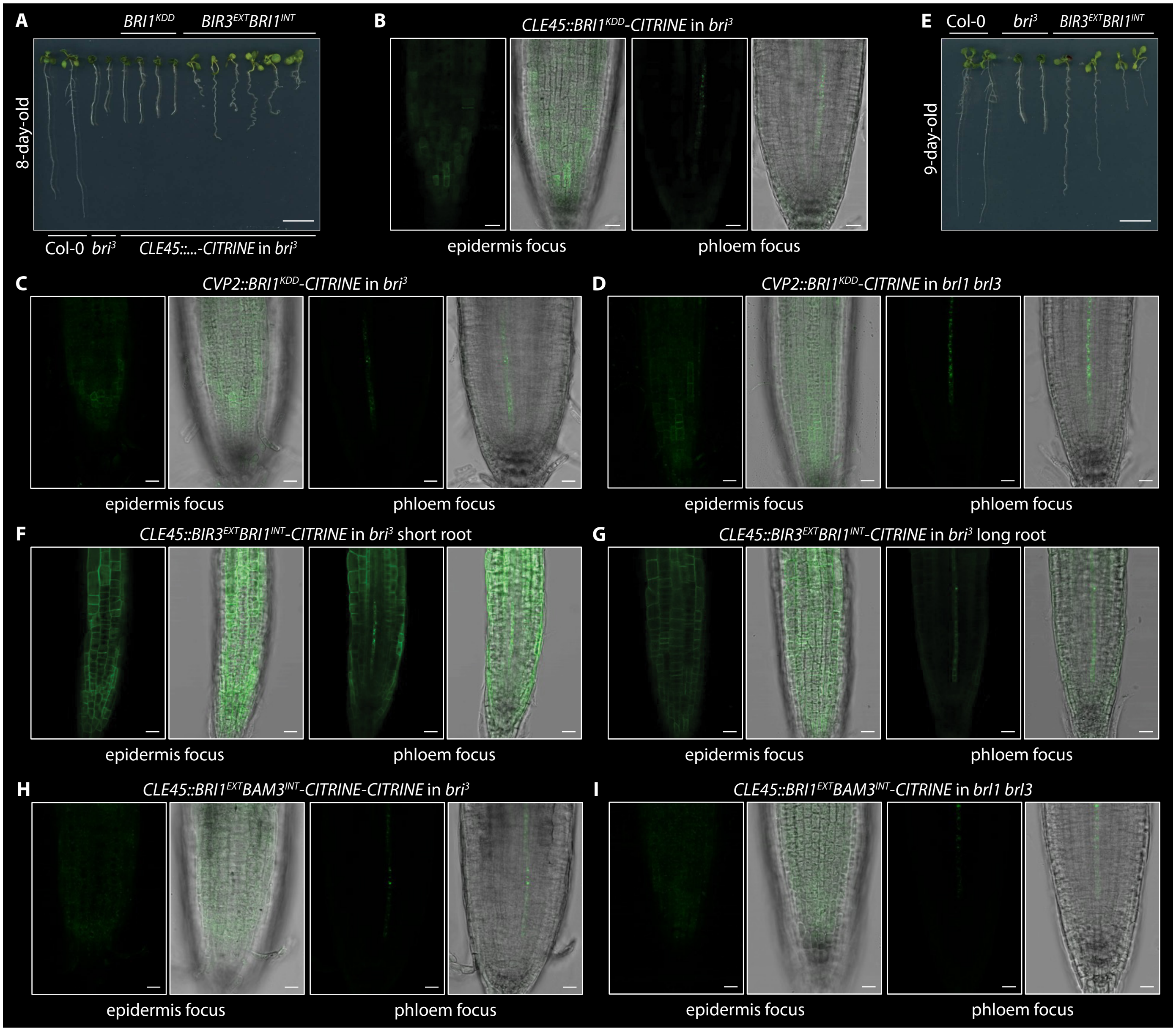

fig. S5. Active BRI1 kinase domain is necessary and sufficient for *bri*^{3} complementation. (**A**) Representative 8-day-old *bri*^{3} seedlings expressing CITRINE fusion proteins with an inactive (BRI1^{KDD}-CITRINE) or a constitutively active (BIR3^{EXT}BRI1^{INT}) BRI1 kinase domain under control of the phloem sieve element-specific *CLE45* promoter, compared to controls. (**B-D**) Confocal microscopy images of root meristems from *bri*^{3} mutant seedlings (B-C) or morphologically wildtype *brl1 brl3* double mutant seedlings (D) carrying either a *CLE45::BRI1*^{KDD}*-CITRINE* (B) or a *CVP2::BRI1*^{KDD}*-CITRINE* (C-D) transgene. (**E**) Representative 9-day-old *bri*^{3} seedlings carrying a *CLE45::BIR3*^{EXT}*BRI1*^{INT}*-CITRINE* transgene, compared to controls, illustrating the phenotypic range observed (also see (A)). (**F-G**) Confocal microscopy images of root meristems from *bri*^{3} mutants expressing the *CLE45::BIR3*^{EXT}*BRI1*^{INT}*-CITRINE* transgene across the phenotypic range illustrated in (E). Note the ubiquitous fusion protein signal. (**H-I**) Confocal microscopy images of root meristems from a 5-day-old *bri*^{3} mutant (H) or morphologically wildtype *brl1 brl3* double mutant (I) expressing a *CLE45::BRI1*^{EXT}*BAM3*^{INT}*-CITRINE* transgene. Size bars are 20µm in microscopy images and 1cm in seedling images.

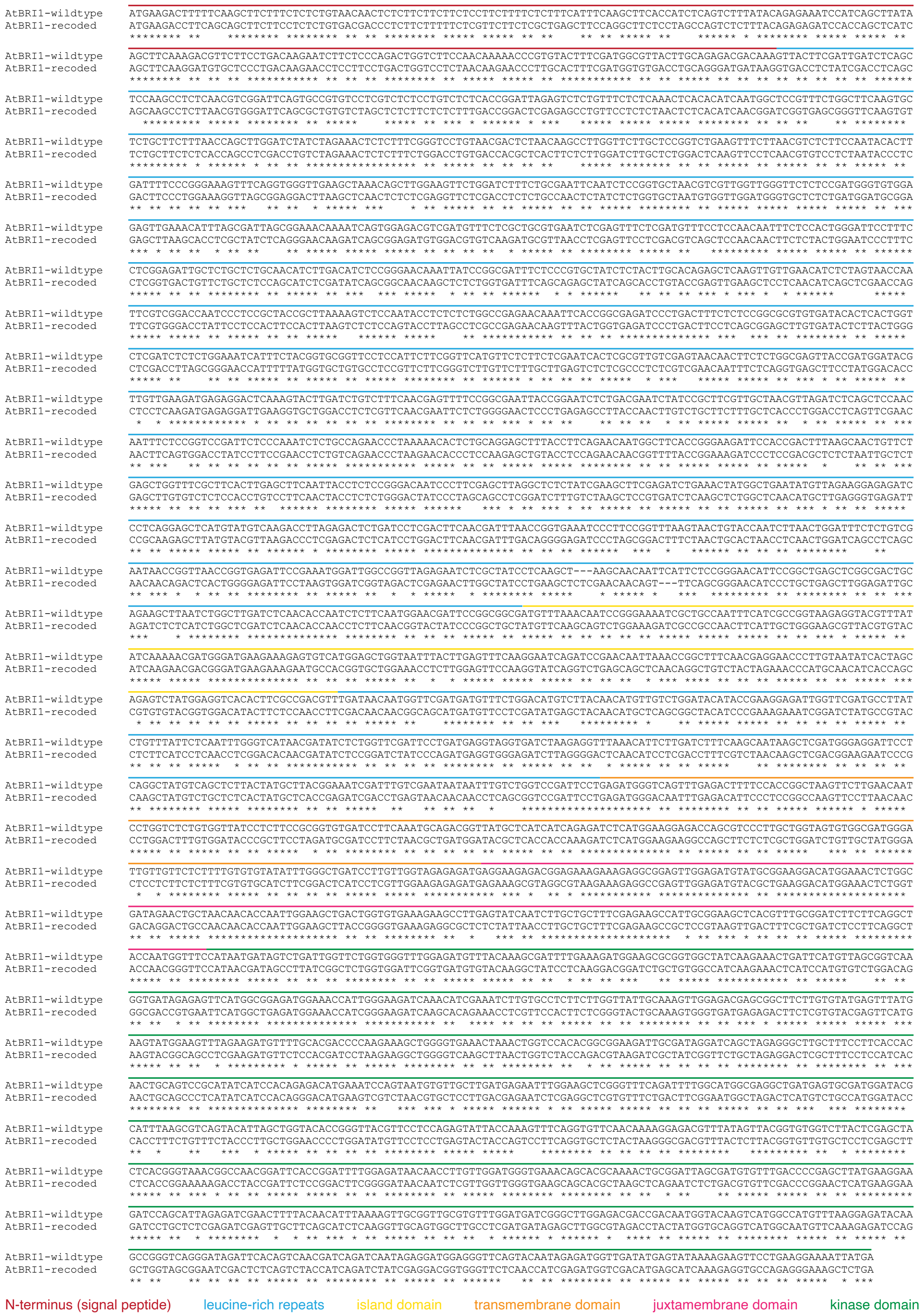

fig. S6. Recoded *BRI1* gene body sequence. Alignment of the native *BRI1* coding sequence and the recoded version, with the corresponding protein domains indicated. Note that the coding sequence of *BRI1* and other brassinosteroid receptor genes is not interrupted by any introns.

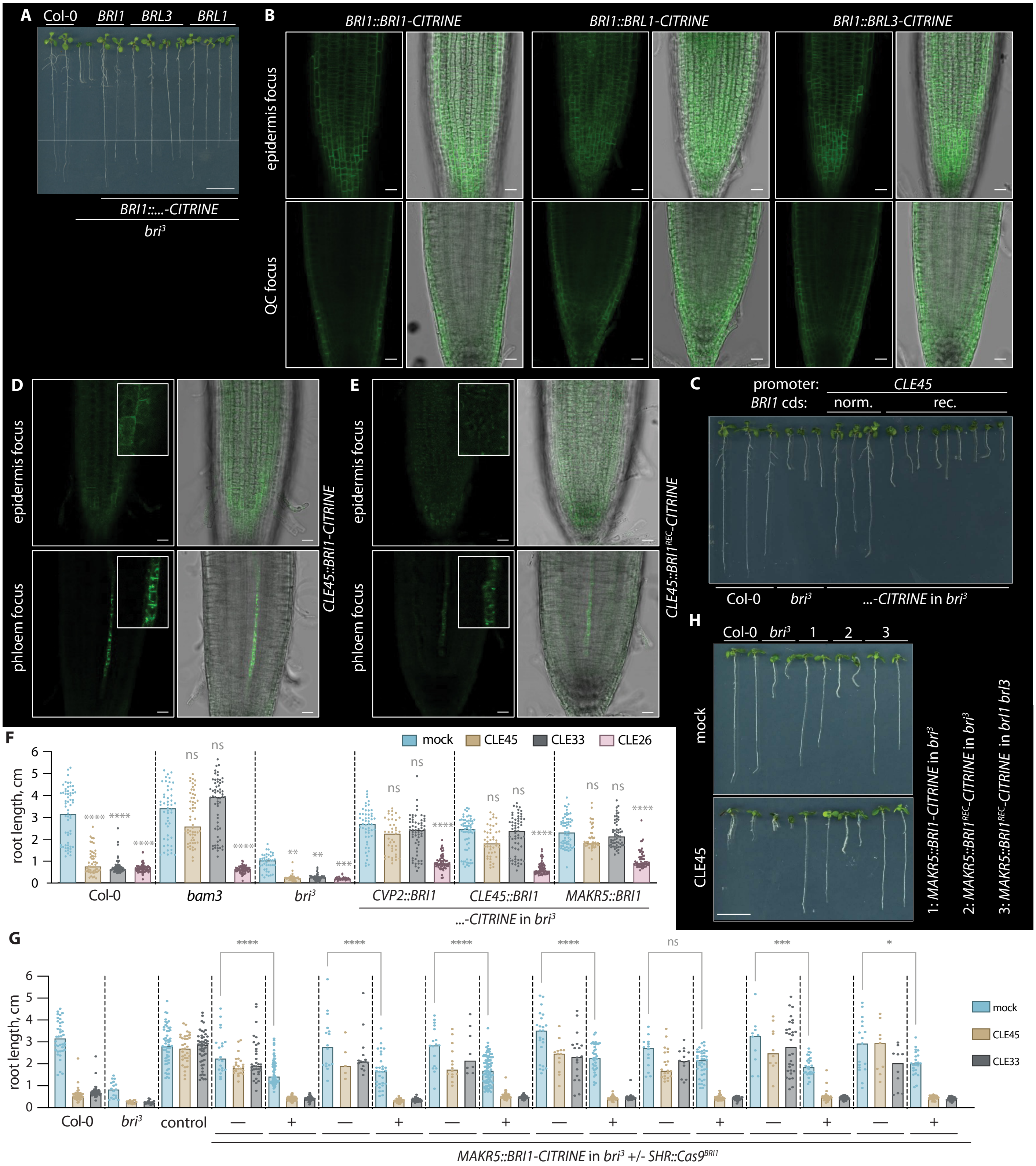

fig. S7. Recoded *BRI1* transgenes produce functional BRI1 fusion protein. (**A**) Representative 9-d-old bri^3 seedlings expressing brassinosteroid receptors under control of the native *BRI1* promoter, compared to wildtype and bri^3 controls. (**B**) Confocal microscopy images of root meristems from seedlings shown in (A). (**C**) Representative 8-d-old bri^3 or morphologically wildtype *brl1 brl3* double mutant seedlings carrying different *BRI1-CITRINE* transgenes made with the genuine (norm.) or recoded (rec.) *BRI1* coding sequence (cds), compared to wildtype and bri^3 controls. (**D-E**) Confocal microscopy images of root meristems from seedlings expressing BRI1-CITRINE or BRI1^{REC}-CITRINE fusion protein under control of the *CLE45* promoter in bri^3 mutants, illustrating the absence of epidermal BRI1^{REC}-CITRINE signal. (**F-G**) Root growth quantification for 7-d-old seedlings of the indicated genotypes grown on mock or in the presence of 20nM of the indicated CLE peptides as compared to controls. (F) The *bam3* mutant is specifically insensitive to CLE45 and the control peptide CLE33, but sensitive to the control peptide CLE26. bri^3 lines complemented with *BRI1-CITRINE* expressed under control of phloem (pole)-specific promoters behave similarly. (G) A bri^3 line complemented with *BRI1-CITRINE* expressed under control of the phloem pole-specific *MAKR5* promoter (control) was combined with tissue-specific CRISPR/Cas9 *BRI1* knockout using the stele-specific *SHR* promoter, several independent lines are shown. Statistically significant differences (asterisks) between seedlings carrying the CRISPR/Cas9 construct and their segregating non-transgenic siblings were determined by ordinary one-way ANOVA, $p<0.001$. (**H**) Representative 8-d-old mock (top) or CLE45 peptide-treated (20nM) (bottom) bri^3 or morphologically wildtype *brl1 brl3* double mutant seedlings expressing BRI1-CITRINE or BRI1^{REC}-CITRINE fusion protein under control of the *MAKR5* promoter, compared to controls. Note that the mock panel is reproduced from Figure 5E. Size bars are 20µm in microscopy images and 1cm in seedling images.

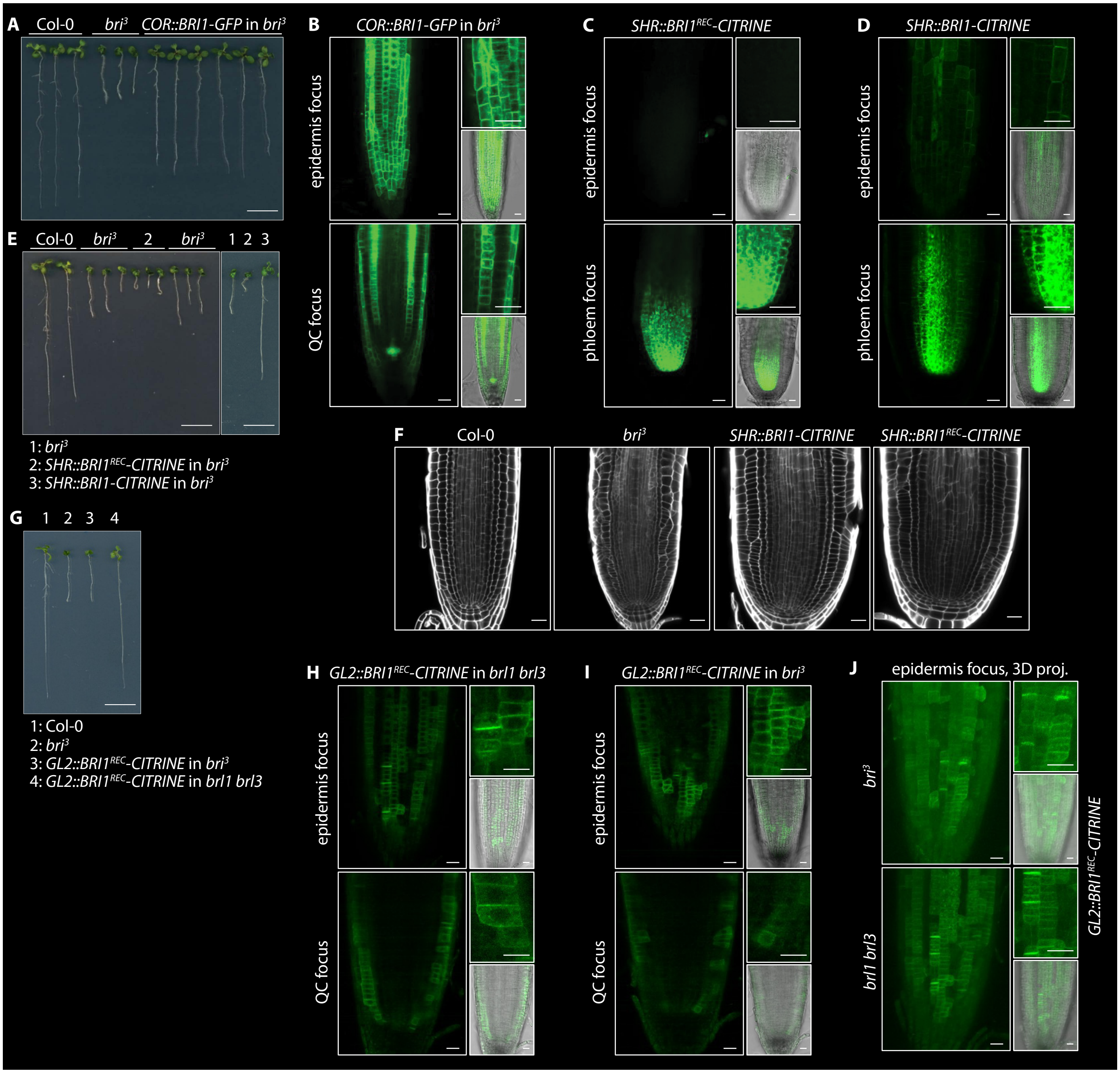

fig. S8. BRI1-CITRINE, but not BRI1^{REC}-CITRINE, rescues *bri^3* when expressed with a stele- or cortex-specific promoter. (**A**) Representative 8-day-old *bri^3* seedlings complemented with a BRI1-GFP fusion protein expressed under control of the cortex-specific *COR* promoter, compared to controls. (**B**) Confocal microscopy images of a root meristem from seedlings shown in (A). Note the presence of a background marker gene expressed in the quiescent center (QC). (**C-D**) Confocal microscopy images of a root meristem from a *bri^3* mutant expressing BRI1-CITRINE or BRI1^{REC}-CITRINE fusion protein under control of the stele-specific *SHR* promoter. (**E**) Representative 8-day-old seedlings expressing BRI1-CITRINE or BRI1^{REC}-CITRINE fusion protein under control of the *SHR* promoter in *bri^3* mutants, compared to controls. (**F**) Confocal microscopy images of calcofluor-stained root meristems from *bri^3* mutants and their counterparts expressing either BRI1-CITRINE or BRI1^{REC}-CITRINE fusion protein under control of the *SHR* promoter. Right panels reproduced from Figure 5. (**G**) Representative 8-day-old seedlings expressing BRI1^{REC}-CITRINE under control of the *GL2* promoter, compared to wildtype and *bri^3* controls. (**H-J**) Confocal microscopy images of root meristems from the indicated genotypes. QC = quiescent center. 3D projections focused on the epidermis are shown in (J). Size bars are 20μm in microscopy images and 1cm in seedling images.

Supplemental auxiliary files

Data S1 scRNAseq analyses.

Data S2 DNA sequences.
